# Nose profile morphology and accuracy study of nose profile estimation method in Scottish subadult and Indonesian adult populations

**DOI:** 10.1007/s00414-017-1758-4

**Published:** 2017-12-19

**Authors:** Erli Sarilita, Christopher Rynn, Peter A. Mossey, Sue Black, Fahmi Oscandar

**Affiliations:** 1 Centre for Anatomy and Human Identification, University of Dundee, Dow Street DD15EH, Dundee, Scotland UK; 2 School of Dentistry, University of Dundee, Dundee, Scotland UK; 3 Faculty of Dentistry, Universitas Padjadjaran, Kabupaten Sumedang, Jawa Barat Indonesia

**Keywords:** Craniofacial reconstruction, Forensic anthropology, Nose morphology, Nose profile estimation

## Abstract

This study investigated nose profile morphology and its relationship to the skull in Scottish subadult and Indonesian adult populations, with the aim of improving the accuracy of forensic craniofacial reconstruction. Samples of 86 lateral head cephalograms from Dundee Dental School (mean age, 11.8 years) and 335 lateral head cephalograms from the Universitas Padjadjaran Dental Hospital, Bandung, Indonesia (mean age 24.2 years), were measured. The method of nose profile estimation based on skull morphology previously proposed by Rynn and colleagues in 2010 (FSMP 6:20–34) was tested in this study. Following this method, three nasal aperture-related craniometrics and six nose profile dimensions were measured from the cephalograms. To assess the accuracy of the method, six nose profile dimensions were estimated from the three craniometric parameters using the published method and then compared to the actual nose profile dimensions.

In the Scottish subadult population, no sexual dimorphism was evident in the measured dimensions. In contrast, sexual dimorphism of the Indonesian adult population was evident in all craniometric and nose profile dimensions; notably, males exhibited statistically significant larger values than females. The published method by Rynn and colleagues (FSMP 6:20–34, 2010) performed better in the Scottish subadult population (mean difference of maximum, 2.35 mm) compared to the Indonesian adult population (mean difference of maximum, 5.42 mm in males and 4.89 mm in females).

In addition, regression formulae were derived to estimate nose profile dimensions based on the craniometric measurements for the Indonesian adult population. The published method is not sufficiently accurate for use on the Indonesian population, so the derived method should be used. The accuracy of the published method by Rynn and colleagues (FSMP 6:20–34, 2010) was sufficiently reliable to be applied in Scottish subadult population.

## Introduction

In order to establish the identity of severely damaged human remains, in which the primary methods such as dental, DNA and finger print identification have failed, craniofacial reconstruction may offer a solution in facilitating familial recognition. Thereafter, the primary identification methods can be attempted again to reaffirm the identity [1–4]. To achieve a recognisable depiction of the face in life, a sound knowledge of craniofacial skeletal and muscular anatomy is crucial because the relationship between the two contributes to the uniqueness of each individual face. Currently, the University of Dundee utilises a combination method for facial reconstruction, merging both facial approximation (average soft tissue depths (ASTD) and guidelines for facial feature estimation derived by regression analysis) and facial reconstruction (sculpting of facial musculature, employment of anatomical pattern between skull and facial features) [1, 4].

Anatomical patterning is a term used here to describe where the shape of the individual skull overrules the ASTD pattern. This occurs in three main areas. Firstly, the bone beneath the superficial masticatory muscles (the ramus of the mandible beneath masseter and the temporal fossa beneath temporalis). If these areas are more deeply concave than usual, with a raised zygomatic arch, it dictates bulkier muscles, hence deeper than average tissue. Secondly, if areas such as the brow ridge or the zygomatic bones are more prominent than usual, slightly deeper than average tissue should be applied [5]. Even if overestimated, this would have the effect of slight caricaturisation, likely, if anything, improving the recognisability of the facial reconstruction. Thirdly, regarding the mouth, dental occlusion or malocclusion has a measurable effect on the tissue depth, with a skeletal class II overjet producing deeper tissue below the mouth [6] and conversely a skeletal class III anterior cross-bite (protrusive mandible) producing deeper tissue above the mouth, as the muscles around the mouth react to each malocclusion to preserve lip competence in everyday life.

Although the literature is very limited, researchers have attempted to provide guidelines that will permit the accurate estimation of soft tissue anatomy for the nose from the skulls of both adults and children [1, 7–13]. The method proposed by Rynn and colleagues [1] which was derived from Caucasian adult population provided simple steps utilising linear measurements and regression equations. In the matter of practicality, this is beneficial for craniofacial reconstruction practitioners due to difficulties experienced in measuring angles directly from the physical skull. In 2013 [14], a study by Mala et al. compared two nose reconstruction methods in 86 cephalograms from Central European adults, which resulted in improved accuracy and practical applicability for the Rynn method [1].

The aim of this study was to investigate the accuracy of the Rynn method [1] on samples from different ethnic groups and from different age ranges.

The objectives of this study:

1. To test for sexual dimorphism in nasal dimensions
2. To quantify the error of the published method [1] by comparing estimated to actual nose profile dimensions
3. If necessary, to derive more accurate regression formulae for use on different populations and different age groups.

## Materials and methods

Eighty-six lateral cephalograms were collected from the archive of orthodontic patients at the Dental School University of Dundee, Scotland. The East of Scotland Research Ethics Service granted the ethical approval for this study in December 2015 (REC ref.: 15/ES/0186). The images were derived from orthodontic patients prior to orthodontic treatment (41 males and 45 females; age range, 8–16 years; average age, 11.85 ± 2.07 years). The cephalograms were digitised using an Epson Perfection V600 scanner.

Three hundred thirty-five lateral cephalograms were selected from the archive of the Radiology Clinic, Universitas Padjadjaran Dental Hospital, Bandung, Indonesia, in the form of bitmap images (.BMP). The Health Research Ethics Committee, Universitas Padjadjaran, provided the ethical approval for this study (Number 812/UN6.C1.3.2/KEPK/PN/2015). The samples were derived from orthodontic patients prior to orthodontic treatment (83 males and 252 females; age range, 17–51 years; average age, 24.17 ± 5.81 years; adult East Asian Mongoloid).

The collected samples of the two populations were representative of the natural population of the radiography archive. Patients with facial syndrome were already excluded from the sample.

**Fig. 1:**
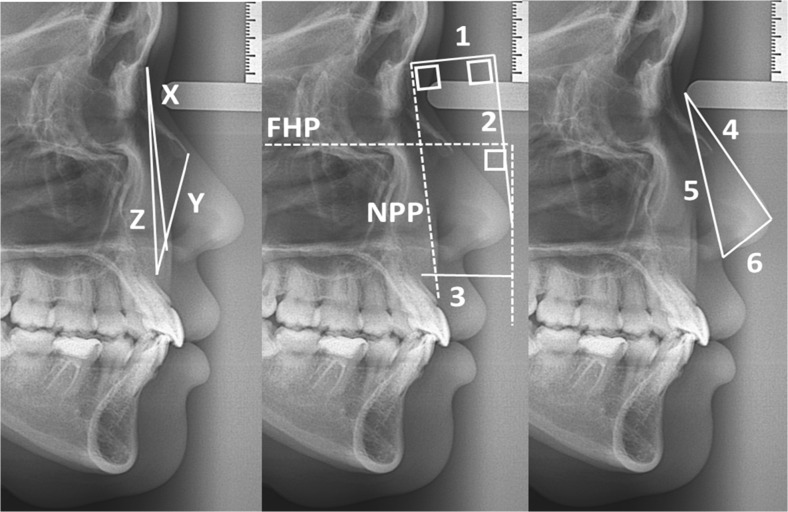
Craniometric measurements (*X*, *Y*, *Z*) and nose profile dimension (1 = PA; 2 = PV; 3 = PFHP, 4 = NL; 5 = NH; 6 = ND; FHP = Frankfurt horizontal plane; NPP = nasion-prosthion plane)

Using Adobe Photoshop CS5.1, the images were rescaled following the millilitre measurement scale in the image. Ruler tool and image size option (pixels per millimetres) were used in rescaling the images. The enlargement of the X-ray compared to the real cranial size was less than 10%. Thus, the image adjustment effect did not impair the image quality, and in turn, image quality did not compromise point identification and therefore these measurements can be deemed to be accurate. Distances between nasion-acanthion (*X*), rhinion-subspinale (*Y*) and nasion-subspinale (*Z*) were measured (Fig. 1). Following the nasal prediction formulae proposed by Rynn and colleagues [1], six dimensions of the estimated nose profile were calculated using *X*, *Y* and *Z* measurements of the nasal aperture: anterior pronasal projection (PA), pronasal projection height (PV), pronasal projection in the Frankfurt horizontal plane (PFHP), nasal length (NL), nasal height (NH) and nasal depth (ND) (Fig. 1). Inter-observer and intra-observer studies were conducted to test the reliability and reproducibility of the measurements. Two practitioners with both dentistry and human anatomy qualifications performed the digital measurements. Statistical analyses were performed using SPSS V22 (IBM Statistical Package for Social Sciences, Version 22).

## Results

### Inter- and intra-observer study

To assess the reliability and repeatability of the measurements, intra- and inter-observer studies were performed. Intraclass correlation coefficient (ICC) tests were used to analyse the results. All variables showed a very high level of both reliability and repeatability with ICC scores above 0.87 (Table 1).

**Table 1:** Intraclass correlation coefficient (ICC) values for each measurement

	Intra-observer	Inter-observer
*X*	0.988	0.990
*Y*	0.928	0.890
*Z*	0.940	0.931
PA	0.989	0.988
PV	0.985	0.981
PFHP	0.966	0.990
NL	0.942	0.871
NH	0.940	0.879
ND	0.954	0.964

In the Scottish subadult population, an independent *t* test showed that there were no significant differences in both aspects of craniometrics and nose profile dimensions between the sexes (*p* > .05). Therefore, in the following analysis, male and female groups were pooled. In contrast, an independent *t* test verified the presence of sexual dimorphism in the Indonesian adult population in all three craniometrics and six nose profile dimensions (*p* < .001).

Paired sample *t* tests were performed in the Scottish subadult population to determine whether there was a statistically significant mean difference between the actual and estimated nose profile dimensions using the published method [1] (Table 2). The published method [1] produced statistically significant mean differences between the actual and the estimated measurements in PV, NH and ND dimensions. The percentages of mean difference / actual mean ratio ranged from 0.83 to 14.05%. However, the only estimated variable which produced a value greater than 1.5% was nasal depth (ND).

**Table 2:** Comparison of the actual and estimated nose profile dimension using the published method in the Scottish subadult population

	PA	PV	PFHP	NL	NH	ND
Actual mean (mm)	25.96	39.86	27.16	42.38	49.00	16.74
Standard deviation	2.80	3.34	3.42	4.25	4.05	1.84
Estimated mean (mm)	25.75	40.45	26.77	41.74	49.72	19.10
Standard deviation	2.77	2.71	3.10	2.49	2.36	1.46
Mean difference (mm)	0.21	− 0.59*	0.39	0.63	− 0.72*	− 2.35*
SD difference (mm)	2.82	2.14	3.26	3.18	2.86	1.84
Paired *t* test, *p* value	0.48	0.01	0.27	0.07	0.02	0.00
Correlation	0.49*	0.77*	0.50*	0.67*	0.72*	0.40*
Correlation *p* value	0.00	0.00	0.00	0.00	0.00	0.00
(Mean difference / actual value) × 100	0.83	1.48	1.44	1.49	1.48	14.05

*p < .05

In the Indonesian adult population, the published method produced statistically significant mean differences between the actual and the estimated measurements in all six nasal dimensions in both male and female groups (Tables 3 and 4). The percentage of mean difference − actual mean ratio ranged from 2.86 to 16.66% in males and 3.66 to 15.47% in females. Ten percent or greater variation was exhibited in three variables for males (PA, NH and ND) and for the same three variables in females.

**Table 3:** Comparison of the actual and estimated nose profile dimension using the published method in Indonesian males (*p < .05)

	PA	PV	PFHP	NL	NH	ND
Actual mean (mm)	24.11	46.91	27.31	44.01	49.49	16.96
Standard deviation	3.21	4.15	2.79	3.50	5.08	1.92
Estimated mean (mm)	27.11	45.57	28.30	46.60	54.91	19.79
Standard deviation	3.16	3.12	3.55	4.06	4.25	1.53
Mean difference (mm)	3.01*	− 1.34*	0.99*	2.59*	5.42*	2.83*
SD difference (mm)	3.05	2.46	2.99	4.43	2.73	2.30
Paired *t* test, *p* value	0.00	0.00	0.00	0.00	0.00	0.00
Correlation	0.54*	0.81*	0.58*	0.32*	0.84*	0.13*
Correlation *p* value	0.00	0.00	0.00	0.00	0.00	0.25
(Mean difference / actual value) × 100	12.48	2.86	3.61	5.89	10.95	16.66

*significant difference at the 0.05 level

**Table 4:** Comparison of the actual and estimated nose profile dimension using the published method in Indonesian females (*p < .05)

	PA	PV	PFHP	NL	NH	ND
Actual mean (mm)	20.89	44.19	23.54	40.35	46.59	15.92
Standard deviation	2.18	2.73	2.23	3.13	3.25	1.47
Estimated mean (mm)	24.13	42.57	24.95	44.00	51.48	18.14
Standard deviation	1.89	2.40	2.11	2.17	1.85	1.14
Mean difference (mm)	3.23*	− 1.62*	1.41*	3.66*	4.89*	2.22*
SD difference (mm)	2.09	1.63	2.32	2.90	2.77	1.39
Paired *t* test, *p* value	0.00	0.00	0.00	0.00	0.00	0.00
Correlation	0.48*	0.81*	0.43*	0.45*	0.53*	0.45*
Correlation *p* value	0.00	0.00	0.00	0.00	0.00	0.00
(Mean difference / actual value) × 100	15.47	3.66	6.00	9.06	10.49	13.97

*Significant difference at the 0.05 level

Overall, the published method produced less error for nose profile estimation for the Scottish subadult population compared to the Indonesian adult population (Tables 2–4). The published method yielded error ranging from the following: 0.21–2.35 mm in Scottish subadults, 0.99–5.42 mm in Indonesian male adults and 1.41–4.89 mm in Indonesian female adults. To illustrate the degree of accuracy of the published method when applied to each population, the percentage of cases within a 2-mm error and within a 5% error were tabulated (Table 5).

**Table 5:** The percentage of individuals whose percentage of nose profile dimension were reconstructed using the published method within a 2-mm error and within a 5% error from the actual dimension

		PA	PV	PFHP	NL	NH	ND
Scottish subadults	Total sample (*n* = 86)						
	% of cases within a 2-mm error	52	62	50	43	57	35
	% of cases within a 5% error	41	62	35	45	69	16
	5% error (mm)	± 1.3	± 2.0	± 1.4	± 2.1	± 2.4	± 0.8
	Males (*n* = 41)						
	% of cases within a 2-mm error	46	63	46	49	59	29
	% of cases within a 5% error	39	63	27	51	68	10
	5% error (mm)	± 1.3	± 2.0	± 1.3	± 2.1	± 2.5	± 0.8
	Females (*n* = 45)						
	% of cases within a 2-mm error	58	60	53	38	56	40
	% of cases within a 5% error	42	60	44	40	69	22
	5% error (mm)	± 1.3	± 2.0	± 1.4	± 2.1	± 2.4	± 0.8
Indonesian adults	Total sample (*n* = 335)						
	% of cases within a 2-mm error	29	58	55	28	13	41
	% of cases within a 5% error	15	64	34	28	16	13
	5% error (mm)	± 1.1	± 2.2	± 1.2	± 2.1	± 2.4	± 0.8
	Males (*n* = 80)						
	% of cases within a 2-mm error	36	64	55	34	10	41
	% of cases within a 5% error	20	67	45	39	11	8
	5% error (mm)	± 1.2	± 2.2	± 1.5	± 2.1	± 2.5	± 0.9
	Females (*n* = 255)						
	% of cases within a 2 mm error	27	56	55	26	13	41
	% of cases within 5% error	13	61	32	28	18	14
	5% error (mm)	± 1.0	± 2.1	± 1.1	± 2.1	± 2.4	± 0.8

### New regression formulae for Indonesian population

Based on Table 5, less error occurred when the published method was applied to the Scottish subadult population compared to when it was applied to the Indonesian adult population. Therefore, further analysis was required to find more appropriate nose profile estimation regression formulae for the Indonesian adult population.

A Pearson correlation test was conducted to evaluate the relationship between craniometrics and nose profile dimensions in the male and female Indonesian groups. The highest correlation between craniometric dimensions and nose profile variables were chosen to represent the independent variables in the next linear regression analysis (Table 6). The highest correlation values were produced by different craniometrics dimension in NH and ND for male and female groups. Hence, the independent variables which were used in the linear regression for NH and ND were separated by sex.

**Table 6:** List of the highest correlation between each nose profile parameters to the craniometrics variable

Nose profile parameters	Male		Female	
	Independent variable	Pearson correlation	Independent variable	Pearson correlation
PA	*Y*	0.550*	*Y*	0.476*
PV	*X*	0.844*	*X*	0.806*
PFHP	*Y*	0.597*	*Y*	0.432*
NL	*X*	0.456*	*X*	0.513*
NH	*Z*	0.845*	*X*	0.566*
ND	*Z*	0.632*	*Y*	0.451*

*p < .001

Linear regressions were run for each pair of nose profile—craniometrics variable listed in Table 6. Except for NH and ND nose profile dimensions, the male and female population were pooled based on the craniometrics variables similarity of the highest correlation. To summarise, the regression formulae derived from the Indonesian adult population and the published method [1] regression formulae were tabulated (Table 7). The degree of concurrence between actual measurements and measurements estimated by the published method [1], and between actual measurements and measurements estimated by the derived method, was calculated using the intraclass correlation coefficient (ICC). The regression formulae derived from the Indonesian adult population improved concurrence across the board (Table 7). The derived method decreased the percentage of mean difference − actual mean ratio to a range of 0.59–4.92% in males (Table 8) and 0.05–1.96% in females (Table 9).

**Table 7:** Nose profile estimation formulae generated by Indonesia adult population and the published method

Nose profile dimension	Published method		ICC of actual vs published method	Derived method		ICC of actual vs derived method
	Male	Female		Male	Female	
PA	0.83 × *Y* – 3.5		0.541*	0.57 × *Y* + 2.33		0.725*
PV	0.90 × *X* – 2		0.846*	0.88 × *X* + 0.68		0.906*
PFHP	0.93 × *Y* – 6		0.727*	0.58 × *Y* + 4.55		0.730*
NL	0.74 × *Z* + 3.5		0.477*	0.66 × *X* + 7.77		0.685*
NH	0.78 × *Z* + 9.5	0.79 × *Z* + 3.31	0.515*	0.79 × *Z* + 3.74	0.69 × *X* + 12.36	0.830*
ND	0.40 × *Y* + 5	0.23 × *Z* + 3.71	0.317*	0.22 × *Z* + 4.02	0.29 × *Y* + 6.24	0.655*

*p < .001

**Table 8:** Comparison of the actual and estimated nose profile dimension using the derived method in Indonesian males (*p < .05)

	PA	PV	PFHP	NL	NH	ND
Actual mean (mm)	24.11	46.91	27.31	44.01	49.49	16.96
Standard deviation	3.21	4.15	2.79	3.50	5.08	1.92
Estimated mean (mm)	23.38	47.29	25.97	42.72	49.78	16.84
Standard deviation	2.16	3.01	2.20	2.25	4.32	1.20
Mean difference (mm)	− 0.72*	0.38	− 1.34*	− 1.28*	0.29	− 0.12
SD difference (mm)	2.71	2.28	2.30	3.18	2.72	1.49
Paired t test *p* value	0.02	0.13	0.00	0.00	0.33	0.46
Correlation	0.55*	0.84*	0.60*	0.46*	0.84*	0.63*
Correlation *p* value	0.00	0.00	0.00	0.00	0.00	0.00
(Mean difference / actual value) × 100	3.01	0.81	4.92	2.91	0.59	0.72

*Significant difference at the 0.05 level

**Table 9:** Comparison of the actual and estimated nose profile dimension using the derived method in Indonesian females (*p < .05)

	PA	PV	PFHP	NL	NH	ND
Actual mean (mm)	20.89	44.19	23.54	40.35	46.59	15.92
Standard deviation	2.18	2.73	2.23	3.13	3.25	1.47
Estimated mean (mm)	21.30	44.31	23.85	40.49	46.57	15.89
Standard deviation	1.30	2.35	1.32	1.76	1.84	0.66
Mean difference (mm)	0.41*	0.12	0.31*	0.14	− 0.03	− 0.03
SD difference (mm)	1.93	1.63	2.05	2.69	2.68	1.31
Paired *t* test, *p* value	0.00	0.25	0.02	0.40	0.88	0.75
Correlation	0.48*	0.81*	0.43*	0.51*	0.57*	0.45*
Correlation *p* value	0.00	0.00	0.00	0.00	0.00	0.00
(Mean difference / actual value) × 100	1.96	0.27	1.33	0.36	0.05	0.17

*Significant difference at the 0.05 level

**Fig. 2:**
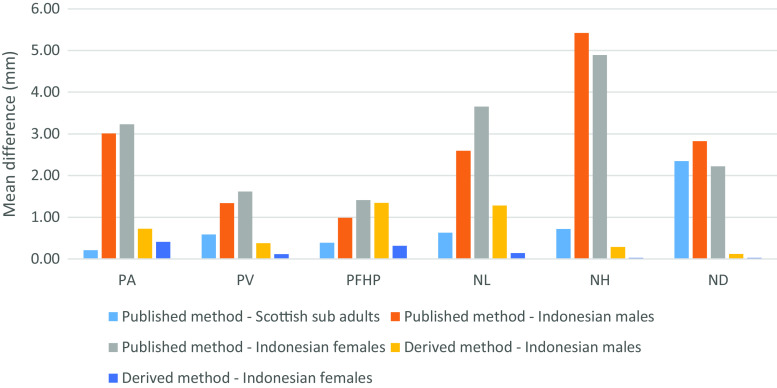
Bar chart of mean differences between two methods in absolute values

The published method [1] generated less error in the Scottish subadult population than when applied to the Indonesian adult population (Fig. 2). Overall, the error produced by the method derived from the Indonesian adult population was lower than the error produced by published method [1] in the Indonesian adult population, both males and females.

## Discussions

The nose profile study requires samples of clear images of soft tissue profile as well as the underlying hard tissue shape in standardised position, which is best represented by cephalometric images. The exposure to non-therapeutic ionising radiation in contemporary medical practice is unacceptable and unethical particularly when it is possible to acquire the information from previously recorded and archived cephalograms. Therefore, our study design enabled us to acquire a large population of healthy individuals without exposure to non-therapeutic X-ray radiation by the use of orthodontic patients’ cephalograms readily available in archives. This will incline the results towards the nose profile measurements in the general malocclusion population as well as the age range and sex of the orthodontic patients trending in the sample source area. Hence, malocclusions, age and sex might contribute as the confounding factors in the result.

The equatorial Indonesian archipelago extends from the islands of Sumatra in the west to the island of Papua in the east. The ancestry of the former is predominantly East Asian and the latter is Australasian. The current study sample was extracted from a dental hospital in West Java which is primarily inhabited by a population of East Asian ancestry. The authors appreciate that the range of sub-ancestral groups within the East Asian umbrella collective will be reflected in the study sample.

In 1994, Hui and colleagues [14] performed a cephalometric study and found that non-cleft patients, who had undergone maxillary osteotomy to treat maxillary hypoplasia, had no significant vertical or horizontal movements of pronasal and subnasal landmarks. In contrast, statistical significant movements were seen in the lip structure. Therefore, this study did not take account of the type of malocclusion of each patient with consideration that it will not affect the nose profile dimensions.

### Scottish subadult and Indonesian adult bony and soft nose morphology comparison

The different stages of maturity attained between the Scottish and Indonesian samples were anticipated because of the different cultural trends in orthodontic treatment between the two countries. The fact that females outnumber males in the Indonesian adult sample was largely due to the societal sex bias in orthodontic intervention towards female adults.

This study does not attempt to compare the statistical difference of nose profile dimensions between the two populations due to the age disparity. However, this is likely to be the major contributor to the difference in reported results. The principal aim of this research was to assess the applicability of the published nose profile estimation method by Rynn and colleagues [1] to the two different populations. In the dimensions of *X*, *Z* and PV, the Scottish subadult group showed the smallest mean dimensions whereas in all others, the smallest dimensions were found in the Indonesian adult females (Table 10).

**Table 10:** Average values of nose morphology between two populations (mm)

	*X*	*Y*	*Z*	PA	PV	PFHP	NL	NH	ND
Scottish subadult	47.20	35.24	51.70	25.96	39.86	27.16	42.38	49.00	16.74
Indonesian adult (m)	52.96	36.93	58.27	24.11	46.91	27.31	44.01	49.49	16.96
Indonesian adult (f)	49.57	33.28	54.73	20.89	44.19	23.54	40.35	46.59	15.92

### Scottish subadult and Indonesian adult accuracy study general comparison

The published method generally produced less error in nose estimation (within a 2-mm error and within a 5% error) in the Scottish subadult population compared to the Indonesian adult population. The similarity in ancestry group between the source sample of the published method [1] and the Scottish population may explain the applicability of the method, despite the difference in age range (published method [1] was derived from adults). The most distinctive error percentage of the published method when compared to the actual Indonesian adults’ nose profile dimension was found in PA (12.48% in male and 15.47% in female) and ND (16.66% in male and 13.97% in female). This could be interpreted as a general difference in the relationship between the nasal aperture and nasal projection, between European and East Asian groups. However, the estimation of ND performed relatively poorly across the board and could be easily excluded from the overall method in practice, in favour of the more accurate formula 3 which estimates a similar dimension, PFHP: the anterior nasal projection from subspinale, in the Frankfurt Horizontal Plane.

### Comparison with other studies regarding nose profile dimension

Three studies from various geographical regions in Europe examining nasal dimensions were compared to the current study (Table 11). The current study of the Scottish subadult population showed comparable results for nose profile dimensions compared to North Italian and Greek children (Table 12). This may be attributable to the three nationalities falling within the same broad Caucasoid ancestry group. In adult samples, NL and NH dimension of the North Italian and Central European groups exhibited similar results whereas Indonesian samples were reported in the lowest values. Moreover, the Central European and Indonesian adults differed markedly in PA, PFHP and ND dimensions. This demonstrated that Central European adult noses may project more anteriorly relative to the nasal aperture, compared to Indonesian adults.

**Table 11:** Sample characteristics of nose profile studies

Author	Ancestry	Sample maturity	Sample size
Ferrario et al. [16]	North Italian	Children	956 (446 males and 510 females)
Halazonetis [17]	Greek	Children	170 (82 males and 88 females)
Present study	Scottish	Children	86 (41 males and 45 females)
Ferrario et al. [16]	North Italian	Adult	101 (57 males and 44 females)
Mala [14]	Central European	Adult	86 (52 males and 34 females)
Present study	Indonesian	Adult	335 (83 males and 252 females)

**Table 12:** Average values comparison of nose profile dimensions between studies

		PA	PV	PFHP	NL	NH	ND
Ferrario et al. [16]*	Male				36.19–43.04	45.86–53.54	
	Female				35.41–42.79	44.05–53.73	
Halazonetis [17]*	Male				42.8	49.4	
	Female				44.2	50.6	
Present Study*	Male	25.68	39.85	27.00	42.12	49.10	16.71
	Female	26.22	39.86	27.31	42.61	48.90	16.77
Ferrario et al. [16]**	Male				49.29	57.59	
	Female				46.37	54.33	
Mala [14]**	Male	32.12	44.92	34.45	48.19	53.81	20.08
	Female	29.20	43.23	31.80	45.89	51.75	19.33
Present Study**	Male	24.11	46.91	27.31	44.01	49.49	16.96
	Female	20.89	44.19	23.54	40.35	46.59	15.92

*Children

**Adults

## Conclusions

This study demonstrates that the relationship between the morphology of the nasal aperture and that of the nose profile is different between the Indonesian population and the predominantly Caucasoid population from which the published method [1] was derived. The published method can be considered sufficiently accurate for prediction of nose dimensions in a Scottish subadult population. It is proposed that the regression equations derived herein from the Indonesian adult population would yield more accurate nasal profile estimations and should be used in the forensic facial reconstruction of unidentified Indonesian individuals and further possibilities to neighbouring countries with same ancestry group. Moreover, the derived method by extension should be tested against the published method [1] on other East Asian adult and subadult groups. Future research will investigate the accuracy of both methods on skulls of related ancestry groups.

## References

[1] Rynn C, Wilkinson CM, Peters HL (2010). Prediction of nasal morphology from the skull. Forensic Sci Med Pathol.

[2] Quatrehomme G, Balaguer T, Staccini P, Alunni-Perret V (2007). Assessment of the accuracy of three-dimensional manual craniofacial reconstruction: a series of 25 controlled cases. Int J Legal Med.

[3] Shahrom AW, Vanezis P, Chapman RC, Gonzales A, Blenkinsop C, Rossi ML (1996). Techniques in facial identification: computer-aided facial reconstruction using a laser scanner and video superimposition. Int J Legal Med.

[4] Wilkinson C (2004). Forensic facial reconstruction.

[5] Gerasimov MM (1955) The reconstruction of the face from the basic structure of the skull. Translated by: Tshernezky W (1975). Moscow, Nauka

[6] Utsuno H, Kageyama T, Uchida K, Yoshino M, Oohigashi S, Miyazawa H, Inoue K (2010). Pilot study of facial soft tissue thickness differences among three skeletal classes in Japanese females. Forensic Sci Int.

[7] George RM (1987). The lateral craniographic method of facial reconstruction. J Forensic Sci.

[8] Prokopec M, Ubelaker DH (2002). Reconstructing the shape of the nose according to the skull. Forensic. Sci Commun.

[9] Stephan CN, Henneberg M, Sampson W (2003). Predicting nose projection and pronasale position in facial approximation: a test of published methods and proposal of new guidelines. Am J Phys Anthropol.

[10] Rynn C, Wilkinson CM (2006). Appraisal of traditional and recently proposed relationships between the hard and soft dimensions of the nose in profile. Am J Phys Anthropol.

[11] Utsuno H, Kageyama T, Uchida K, Deguchi T, Miyazawa H, Inoue K (2008). Estimation of nasal tip position using lateral cephalometric X-ray images in Japanese male children: applications in facial reconstruction. Pediatr Dent J.

[12] Davy-Jow SL, Decker SJ, Ford JM (2012). A simple method of nose tip shape validation for facial approximation. Forensic Sci Int.

[13] Utsuno H, Kageyama T, Uchida K, Kibayashi K, Sakurada K, Uemura K (2016). Pilot study to establish a nasal tip prediction method from unknown human skeletal remains for facial reconstruction and skull photo superimposition as applied to a Japanese male population. J Forensic Legal Med.

[14] Mala PZ (2013). Pronasale position: an appraisal of two recently proposed methods for predicting nasal projection in facial reconstruction. J Forensic Sci.

[15] Hui E, Hägg EUO, Tideman H (1994). Soft tissue changes following maxillary osteotomies in cleft lip and palate and non-cleft patients. J Cranio Maxill Surg.

[16] Ferrario VF, Sforza C, Poggio CE, Schmitz JH (1997). Three-dimensional study of growth and development of the nose. Cleft Palate Craniofac J.

[17] Halazonetis DJ (2007). Morphometric evaluation of soft-tissue profile shape. Am J Orthod Dentofac.

